# Diagnostic Accuracy of Active MMP-8 Point-of-Care Test in Peri-Implantitis

**DOI:** 10.1055/s-0044-1793843

**Published:** 2024-12-10

**Authors:** Ioannis Fragkioudakis, Leonidas Batas, Ioannis Vouros, Dimitra Sakellari

**Affiliations:** 1 Department of Periodontology and Implant Biology, School of Dentistry, Aristotle University of Thessaloniki, Thessaloniki, Greece

**Keywords:** aMMP-8, biomarker, peri-implantitis, dental implants, periodontitis, diagnostic sensitivity, diagnostic specificity

## Abstract

**Objective** This study aimed to evaluate the diagnostic sensitivity and specificity of the active matrix metalloproteinase-8 (aMMP-8) quantitative chairside point-of-care (PoC) lateral flow immunotest for peri-implant diseases, and it sought to correlate aMMP-8 levels with clinical parameters to determine its effectiveness as a biomarker for peri-implantitis.

**Materials and Methods** A cross-sectional study was conducted at the Department of Periodontology and Implant Biology, Aristotle University of Thessaloniki, Greece. Participants included systemically healthy individuals with at least one implant loaded for more than 1 year, who had not received periodontal treatment or antibiotics in the preceding 6 months. Exclusion criteria included diabetes and immune-compromising conditions. Peri-implant sulcular fluid (PISF) samples were collected from the mesiobuccal or distobuccal site of the implant. The quantitative chairside PoC lateral flow immunotest for peri-implant diseases (ImplantSafe test) and ORALyzer digital reader were used to analyze PISF, with results expressed in ng/mL. Clinical parameters such as bleeding on probing (BOP), probing depth (PD), recession (REC), and clinical attachment level (CAL) were measured at six sites per implant using a 15-mm scale periodontal probe.

**Results** No significant differences were found in age, gender distribution, or smoking status between the healthy/mucositis and peri-implantitis groups. The peri-implantitis group showed significantly higher mean percentages of BOP (57.58 ± 31.73 vs. 18.79 ± 24.17), PD (4.59 ± 1.22 mm vs. 2.94 ± 0.78 mm), and CAL (5.21 ± 1.72 mm vs. 3.05 ± 0.81 mm). aMMP-8 levels were also significantly higher in the peri-implantitis group (53.39 ± 49.70 vs. 22.03 ± 32.87). The diagnostic test demonstrated a sensitivity of 81.25% and specificity of 74.07%, with an area under the curve of 79.6%, indicating overall good accuracy in distinguishing between positive and negative conditions.

**Conclusion** The aMMP-8 is a promising biomarker for peri-implantitis, showing elevated levels in affected patients. The aMMP-8 chairside test demonstrates high diagnostic sensitivity and specificity, supporting its use in early detection and monitoring of peri-implant diseases. Further research is needed to establish standardized protocols for its clinical application and to explore its long-term predictive value in implant care.

## Introduction

The increasing focus on biomarkers for diagnosing and monitoring peri-implantitis signifies a pivotal advancement toward more precise and personalized dental care strategies. Among these biomarkers, matrix metalloproteinase-8 (MMP-8), especially in its active matrix metalloproteinase-8 (aMMP-8) form, has emerged as a critical entity due to its significant role in the pathophysiology of both periodontitis and peri-implantitis. MMP-8, an enzyme that degrades collagen, plays a vital role in tissue remodeling and the pathogenesis of inflammatory diseases, making it a key indicator of tissue integrity and disease progression. 1 2

Recent studies have consistently shown elevated activity of MMP-8 in the peri-implant sulcular fluid (PISF) of patients with peri-implantitis. This elevated activity highlights the enzyme's involvement in tissue degradation and active disease processes, thus underscoring its potential as a disease biomarker. 3 4 5

Matrix metalloproteinases (MMPs), including MMP-8, are initially produced as inactive precursors known as latent pro-MMPs. Various agents, including reactive oxygen species, tissue and plasma proteinases, or microbial proteinases, activate these proenzymes. The activation process is crucial, enabling to degrade interstitial collagen at inflammation sites. 1 Elevated levels of aMMP-8, rather than its total or latent forms, are essential for distinguishing healthy tissue from inflammatory conditions such as gingivitis, periodontitis, peri-implant mucositis, and peri-implantitis. 4 6 7 8

The ImplantSafe test leverages monoclonal antibodies to detect aMMP-8 in PISF with diagnostic sensitivity (76–83%) and specificity (96%) as previously reported. 5 9 This test allows for early detection and differentiation between active and quiescent peri-implant sites, enabling the formulation of personalized treatment plans and monitoring strategies. 6

PerioSafe, a point-of-care (PoC) test utilizing lateral flow chromatography, detects active MMP-8 in oral rinse. It is designed to screen for chronic periodontitis, predict disease progression, and monitor treatment efficacy. This test demonstrates a sensitivity and a specificity of 76.5 and 96.7%, respectively. 5 6

Incorporating aMMP-8 into a broader diagnostic and therapeutic framework necessitates an understanding of its correlation with clinical parameters such as bleeding on probing (BOP), probing pocket depth (PPD), and clinical attachment level (CAL). These correlations provide a direct link between biomarker activity and disease severity. However, interpreting aMMP-8 levels should be contextualized within each patient's disease trajectory and treatment response, emphasizing the importance of longitudinal studies to establish the predictive value of aMMP-8 levels over time.

Research by Xanthopoulou et al and Lähteenmäki et al has highlighted the efficacy of aMMP-8 as a biomarker through quantitative PoC chairside lateral flow immunotests. 6 10 Their findings confirm the utility of aMMP-8 in early identification and risk assessment of peri-implant diseases, substantiating its significance in detecting active collagenolysis in peri-implant tissues at various stages of disease progression.

This study aimed to determine the sensitivity and specificity of the aMMP-8 chairside test in the context of peri-implant diseases, demonstrating the significant potential of aMMP-8 in advancing dental care.

## Materials and Methods

### Study Design and Population

This cross-sectional study recruited patients from the Department of Periodontology and Implant Biology at Aristotle University of Thessaloniki, Greece. Eligible participants included patients with at least one implant functionally loaded for more than 1 year, who were systemically healthy. These patients were either periodontally healthy or demonstrated stability in periodontal disease, as defined by Lang and Bartold. 11 Participants were also required to be free from systemic and infectious diseases and have not received periodontal treatment or antibiotics in the preceding 6 months. Exclusion criteria included diabetes and other immune-compromising conditions. The patients were categorized into two groups: those diagnosed with peri-implantitis and those with healthy implants or peri-implant mucositis, according to the current classification. 12 Informed consent was obtained from all participants. The study was approved by the Ethical Committee of the School of Dentistry, Aristotle University of Thessaloniki (115/25-05-21), and it was registered in the ClinicalTrialsGov.gr database under the ID: NCT05711407.

### Sample Collection and Analysis

After initial screening, PISF was collected from the implant site prior to the clinical examination. Prosthetics were removed in order to establish the better quality of the sample. The sample was preferably taken from either the mesiobuccal or distobuccal site of the implant. To prevent saliva contamination, the area was isolated with cotton swabs and any supragingival plaque was removed. The implant was then air dried, and PISF was collected using paper strips. These strips were inserted into the peri-implant sulcus (1–2 mm subgingivally) for 30 seconds. Strips visibly contaminated with blood or saliva were discarded. The collected PISF samples were quantitatively assessed for aMMP-8 levels using the ImplantSafe test (Dentognostics GmbH) and the ORALyzer digital reader, following the manufacturer's instructions, with results expressed in ng/mL.

#### Clinical Examination

Clinical parameters were recorded during the examination phase. The clinical examination included the measurement of BOP, which was recorded as the presence (+) or absence (−) of bleeding, was expressed as a percentage and assessed 30 seconds after a periodontal probe was inserted into the peri-implant pocket. Probing depth (PD) was measured as the distance from the mucosal margin to the bottom of the peri-implant pocket. CAL was noted as the distance from the prosthetic crown shoulder to the bottom of the sulcus or peri-implant pocket. All measurements were taken at six sites per implant using a 15-mm scale periodontal probe (Hu-Friedy CP-15/CP-11.5B Screening Color-Coded Probe) and graded per 1 mm. These measurements were repeated after the removal of the prosthesis. All examinations were performed by the same examiner (I.F.). Intra-examiner reproducibility was calculated during two calibration sessions. Intra-examiner agreement was calculated with an intraclass correlation coefficient (ICC) and showed an agreement of 0.93 (95% CI: 0.89–0.96).

#### Sample Size Calculation

A pilot study estimated the mean and standard deviation of aMMP-8 levels in healthy and peri-implantitis groups. The effect size (Cohen's d) was approximately 0.71, indicating a required sample size of about 30 participants per group to detect significant differences with a power of 0.80 and an alpha level of 0.05.

#### Statistical Analysis

Statistical analysis compared the clinical parameters and biomarker levels between the healthy/mucositis and peri-implantitis groups. Continuous variables were expressed as mean ± standard deviation (SD). Categorical variables were expressed as counts and percentages. Student's *t* -test was used to compare the mean values of continuous variables (e.g., mean % BOP, mean PD, mean CAL, and aMMP-8 ImplantSafe level) between the healthy/mucositis and peri-implantitis groups. The chi-square test was used to compare categorical variables (e.g., sex distribution and smoking status) between the two groups. Mann–Whitney U test was used for non-normally distributed variables. A *p* -value of <0.05 was considered statistically significant. The sensitivity, specificity, the area under the curve (AUC), and the receiver operating characteristic (ROC) were calculated to evaluate the diagnostic performance of the biomarker aMMP-8.

## Results

The demographic characteristics and smoking status of the participants in the healthy/mucositis group ( *n* = 54) and the peri-implantitis group ( *n* = 48) are presented in Table 1 . The mean age of the healthy/mucositis group was 58.22 ± 10.19 years, compared to 60.73 ± 10.54 years in the peri-implantitis group, with no significant difference between the two groups ( *p* = 0.225). Gender distribution was also similar, with 19 males and 35 females in the healthy/mucositis group and 23 males and 25 females in the peri-implantitis group ( *p* = 0.270). The smoking status did not differ significantly between the groups: the healthy/mucositis group included 35 nonsmokers, 9 individuals smoking less than 10 cigarettes per day, and 10 individuals smoking more than 10 cigarettes per day, while the peri-implantitis group included 30 nonsmokers, 6 individuals smoking less than 10 cigarettes per day, and 12 individuals smoking more than 10 cigarettes per day ( *p* = 0.665).

**Table 1:** Demographic data and comparisons between healthy/mucositis and peri-implantitis groups

Parameter	Healthy/mucositis ( *n* = 54)	Peri-implantitis ( *n* = 48)	*p* -value
Age (mean ± SD)	58.22 ± 10.19	60.73 ± 10.54	0.225 a b
Sex	Male: 19 Female: 35	Male: 23 Female: 25	0.270 a b
Smoking (cig/d)	No: 35 <10 cig/d: 9 >10 cig/d: 10	No: 30 <10 cig/d: 6 >10 cig/d: 12	0.665 a b

a Independent sample *t* -test.

b Statistically significant at 0.05 level.

### Clinical Parameters

Table 2 displays the results of the comparisons of clinical parameters between the healthy/mucositis and peri-implantitis groups. The mean percentage of BOP was significantly higher in the peri-implantitis group (57.58 ± 31.73) compared to the healthy/mucositis group (18.79 ± 24.17; *p* < 0.001). The mean PD was also significantly greater in the peri-implantitis group (4.59 ± 1.22 mm) than in the healthy/mucositis group (2.94 ± 0.78 mm; *p* < 0.001). Additionally, the mean CAL was significantly higher in the peri-implantitis group (5.21 ± 1.72 mm) compared to the healthy/mucositis group (3.05 ± 0.81 mm; *p* < 0.001).

**Table 2:** Comparison of clinical parameters of investigated implants between healthy/mucositis and peri-implantitis groups

Parameter	Healthy/mucositis (mean ± SD)	Peri-implantitis (mean ± SD)	*p* -value
Mean % BOP	18.79 ± 24.17	57.58 ± 31.73	<0.001 a b
Mean PD (mm)	2.94 ± 0.78	4.59 ± 1.22	<0.001 a b
Mean CAL (mm)	3.05 ± 0.81	5.21 ± 1.72	<0.001 a b
aMMP-8 ImplantSafe	22.03 ± 32.87	53.39 ± 49.70	<0.001 a b

Abbreviations: BOP, bleeding on probing; CAL, clinical attachment level; PD, probing depth; SD, standard deviation.

a Mann–Whitney U test.

b Statistically significant at 0.05 level.

The aMMP-8 ImplantSafe levels were significantly higher in the peri-implantitis group (53.39 ± 49.70) compared to the healthy/mucositis group (22.03 ± 32.87; *p* < 0.001).

### Clinical Parameters and Demographic Parameters Based on aMMP-8 Levels

Table 3 shows clinical and demographic parameters stratified by aMMP-8 levels (<20 ng/mL and >20 ng/mL). The mean age was similar between participants with aMMP-8 levels <20 ng/mL (59.49 ± 8.79 years) and those with levels >20 ng/mL (59.32 ± 11.74 years; *p* = 0.935). Smoking status did not significantly differ based on aMMP-8 levels: among participants with aMMP-8 levels <20 ng/mL, there were 29 nonsmokers, 7 individuals smoking less than 10 cigarettes per day, and 14 individuals smoking more than 10 cigarettes per day, while among those with levels >20 ng/mL, there were 36 nonsmokers, 8 individuals smoking less than 10 cigarettes per day, and 8 individuals smoking more than 10 cigarettes per day ( *p* = 0.298).

**Table 3:** Clinical and demographic parameters based on aMMP-8 levels (cutoff value 20mg/mL)

Parameter	<20 ng/mL (Mean ± SD)	>20 ng/mL (Mean ± SD)	*p* -value
Age	59.49 ± 8.79	59.32 ± 11.74	0.935
Smoking	No: 29 <10 cig/day: 7 >10 cig/day: 14	No: 36 <10 cig/day: 8 >10 cig/day: 8	0.298
Mean % BOP	26.15 ± 26.41	47.12 ± 37.17	0.002 a b
Mean PD (mm)	3.17 ± 0.91	4.22 ± 1.41	<0.001 a b
Mean CAL (mm)	3.33 ± 1.02	4.75 ± 1.92	<0.001 a b

Abbreviations: BOP, bleeding on probing; CAL, clinical attachment level; PD, probing depth; SD, standard deviation.

a Mann–Whitney U test.

b Statistically significant at 0.05 level.

Significant differences were observed in clinical parameters based on aMMP-8 levels. Participants with aMMP-8 levels >20 ng/mL had a higher mean percentage of BOP (47.12 ± 37.17) compared to those with levels <20 ng/mL (26.15 ± 26.41) ( *p* = 0.002). The mean PD was also greater in the higher aMMP-8 group (4.22 ± 1.41 mm) compared to the lower aMMP-8 group (3.17 ± 0.91 mm; *p* < 0.001). Similarly, the mean CAL was higher in the >20 ng/mL aMMP-8 group (4.75 ± 1.92 mm) than in the <20 ng/mL group (3.33 ± 1.02 mm; *p* < 0.001).

These findings underscore significant differences in clinical parameters between the healthy/mucositis and peri-implantitis groups, as well as notable variations between participants with different aMMP-8 levels.

### Sensitivity and Specificity

**Fig. 1:**
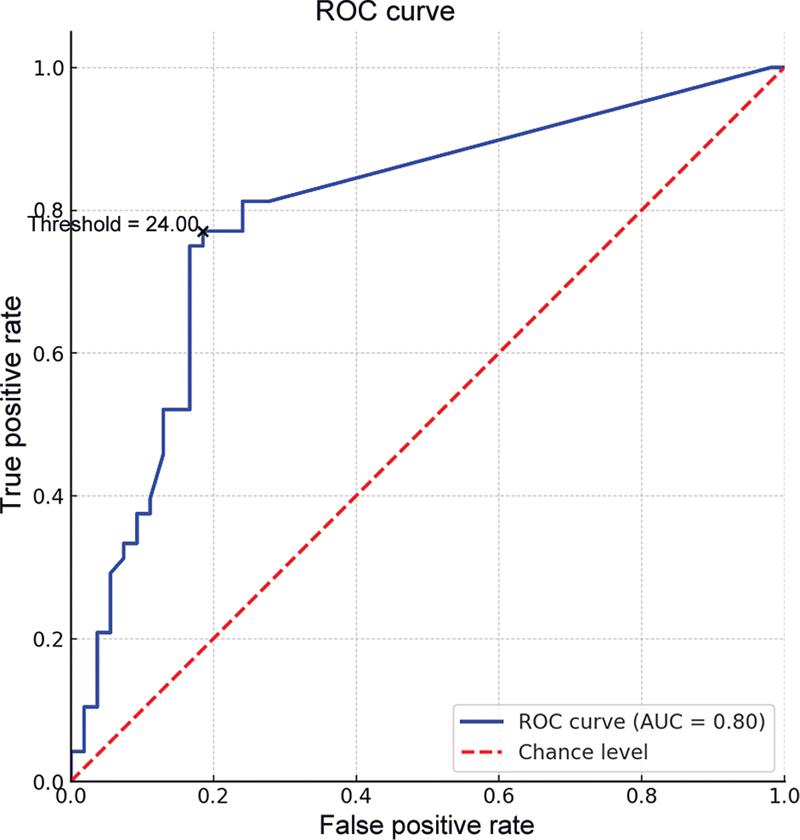
ROC curve for aMMP-8 levels in diagnosing peri-implantitis. AUC, area under the curve; ROC, receiver operating characteristic.

At a cutoff value of 20 ng/mL for aMMP-8 levels in diagnosing peri-implantitis, the AUC was found to be 0.796 ( Fig. 1 ). This indicates an overall good performance of the test. The sensitivity, which measures the ability of the test to correctly identify those with peri-implantitis, was 81.25%. The specificity, which measures the ability of the test to correctly identify those without the condition, was 74.07%. These metrics suggest that using a cutoff value of 20 ng/mL for aMMP-8 provides a reasonable balance between correctly identifying both affected and unaffected individuals.

Consequently, the performance of the MMP-8 diagnostic test for peri-implantitis was evaluated at various cutoff values ranging from 20 to 30 ng/mL. The sensitivity, specificity, positive predictive value (PPV), negative predictive value (NPV), and Youden's index for each cutoff value are presented in Table 4 .

**Table 4:** Performance metrics for different cutoff values of MMP-8

Cutoff value (ng/mL)	Sensitivity (%)	Specificity (%)	PPV (%)	NPV (%)	Youden's index
20	81.25	74.07	73.58	81.63	0.553
21	81.25	75.93	75.00	82.00	0.572
22	79.17	75.93	74.51	80.39	0.551
23	77.08	75.93	74.00	78.85	0.530
24	77.08	81.48	78.72	80.00	0.586
25	75.00	83.33	80.00	78.95	0.583
26	72.92	83.33	79.55	77.59	0.563
27	70.83	83.33	79.07	76.27	0.542
28	70.83	83.33	79.07	76.27	0.542
29	70.83	83.33	79.07	76.27	0.542
30	70.83	83.33	79.07	76.27	0.542

Abbreviations: NPV, negative predictive value; PPV, positive predictive value.

At a cutoff value of 24 ng/mL, the MMP-8 diagnostic test exhibited the highest Youden's index of 0.586, indicating the optimal balance between sensitivity (77.08%) and specificity (81.48%). This suggests that 24 ng/mL is the most effective cutoff value for distinguishing between individuals with and without peri-implantitis based on MMP-8 levels.

## Discussion

The quest for reliable biomarkers in the diagnosis and prognosis of peri-implantitis has become a pivotal focus in contemporary dental research, underscoring a shift toward more precise and personalized oral care strategies. aMMP-8, a collagen-degrading enzyme, stands out as a critical biomarker due to its significant role in the pathophysiology of periodontitis and peri-implantitis. The enzyme's ability to reflect tissue remodeling and inflammatory processes makes it a valuable indicator of peri-implant health. Recent advancements have highlighted the diagnostic potential of aMMP-8 levels in PISF as an early marker of tissue degradation and active disease. 5 7 This study aimed to evaluate the diagnostic sensitivity and specificity of the aMMP-8 PoC test in detecting peri-implant diseases and to correlate these levels with clinical parameters, thereby assessing its effectiveness as a biomarker for peri-implantitis.

Our findings demonstrated significant differences in aMMP-8 levels between healthy/mucositis and peri-implantitis groups, with elevated levels correlating strongly with clinical indicators of disease such as BOP, PD, and CAL. These results support the use of aMMP-8 as a reliable diagnostic tool for early detection and monitoring of peri-implant diseases, potentially leading to more timely and tailored treatment approaches.

The findings of the present study regarding the elevated levels of aMMP-8 in peri-implantitis patients are consistent with results from previous reports. Lähteenmäki et al reported a mean aMMP-8 level of 142.32 ± 117.52 ng/mL in peri-implantitis patients, significantly higher than the 49.25 ± 33.45 ng/mL observed in healthy controls. 13 This aligns with Mäntylä et al, who reported total MMP-8 levels of 80.3 ± 18.6 ng/mL in peri-implantitis patients compared to 10.2 ± 5.4 ng/mL in healthy implants, and Sorsa et al, who reported 75.4 ± 20.1 ng/mL in diseased sites versus 12.3 ± 6.8 ng/mL in healthy ones. 9 14 Additionally, Xanthopoulou et al demonstrated similar trends, with peri-implantitis cases showing aMMP-8 levels at 73.07 ± 43.93 ng/mL, healthy implants at 13.65 ± 7.18 ng/mL, and peri-implant mucositis at 32.33 ± 21.20 ng/mL. 10

The present study reported sensitivity (81.25%) and specificity (74.07%) for the ImplantSafe tests, and these findings are corroborated by other research. Rathnayake et al and Lähteenmäki et al found similar diagnostic performance, with sensitivities of 76 to 83% and specificities of 96% for aMMP-8-based diagnostic tools. The AUC of 0.796 observed in the current study further supports the strong diagnostic capability of these tests, consistent with Lähteenmäki et al, who reported AUCs of 0.880 and 0.883 for quantitative and qualitative (levels >20ng/mL indicate disease) aMMP-8 PoC tests, respectively. 5 13

When compared to traditional clinical parameters such as BOP and PD, aMMP-8 demonstrates superior diagnostic accuracy. Sorsa et al have shown that BOP had a sensitivity of 58% and a specificity of 63%, significantly lower than the sensitivity and specificity of the aMMP-8 test observed in the current study. 9 This suggests that MMP-8 provides a more reliable indication of tissue inflammation and collagen degradation, making it a superior marker for peri-implant health.

Previous research has suggested varying cutoff values, with some studies identifying levels as low as 15.3 ng/mL to be predictive of peri-implantitis onset. For instance, a study reported that implants with a-MMP-8 levels exceeding 15.3 ng/mL had a higher probability of peri-implantitis, underscoring the importance of MMP-8 as a biomarker. 15 In the present study, the recognition of a threshold of 24 ng/mL for aMMP-8 levels as the optimal cutoff for diagnosing peri-implantitis has significant implications for clinical practice. Since the specific higher threshold might better differentiate between those with and without the disease, reducing false positives and improving diagnostic precision. This higher cutoff value aligns with findings from a previous study that highlighted the utility of elevated MMP-8 levels in various inflammatory conditions, including peri-implantitis. 16 The implications of using a 24 ng/mL cutoff are multifaceted. Clinically, it means fewer misdiagnoses and unnecessary treatments, allowing for more targeted therapeutic interventions. This approach not only enhances patient care but also optimizes resource allocation within dental practices.

Despite the promising findings, the variability in reported MMP-8 levels across studies might be attributed to differences in sample collection methods, patient populations, and assay techniques. For example, in the current study sampling procedures and assessment of clinical parameters were performed after removal of the prosthetic components, similar to previous reports. 17 Additionally, the relatively small sample sizes in some studies may limit the generalizability of the results. Therefore, larger, multicenter studies are needed to confirm these findings and establish standardized protocols for MMP-8 measurement. While MMP-8 shows high diagnostic accuracy, it should be integrated with other clinical parameters for a comprehensive assessment of peri-implant health. Longitudinal studies are necessary to determine the predictive value of MMP-8 over time and its role in monitoring treatment outcomes. Additionally, research into the cost-effectiveness and practicality of implementing MMP-8-based diagnostic tools in routine dental practice is essential. Exploring the molecular mechanisms underlying MMP-8 activation and its interaction with other biomarkers could provide deeper insights into the pathogenesis of peri-implantitis and help identify potential therapeutic targets.

## Conclusion

The primary study, along with supporting research, provides further evidence for using MMP-8 as a biomarker in diagnosing and monitoring peri-implantitis. Elevated MMP-8 levels in peri-implantitis patients and the high diagnostic accuracy of tests like ImplantSafe and PerioSafe underscore the enzyme's clinical relevance. The consistent findings across various studies highlight MMP-8 as a superior biomarker compared to traditional clinical parameters. Future research should focus on longitudinal validation, cost-effectiveness, and integrating MMP-8 into a broader diagnostic framework to enhance clinical outcomes in dental care.
